# Predictive Relation between Early Numerical Competencies and Mathematics Achievement in First Grade Portuguese Children

**DOI:** 10.3389/fpsyg.2017.01103

**Published:** 2017-06-30

**Authors:** Lilia Marcelino, Óscar de Sousa, António Lopes

**Affiliations:** ^1^Center of Interdisciplinary Studies in Education and Development, Education Institute, Universidade LusófonaLisbon, Portugal; ^2^Center of Studies in Cognitive Psychology and Learning, Faculty of Psychology, Universidade LusófonaLisbon, Portugal

**Keywords:** early numerical competencies, mathematics achievement, grade 1, short-term longitudinal study, linear regression model

## Abstract

Early numerical competencies (ENC) (counting, number relations, and basic arithmetic operations) have a central position in the initial learning of mathematics, and their assessment is useful for predicting later mathematics achievement. Using a regression model, this study aims to analyze the correlational and predictive evidence between ENC and mathematics achievement in first grade Portuguese children (*n* = 123). The children’s ENC were examined at the point of school entry. Three criterion groups (low, moderate, and high ENC) were formed based on the results of the early numerical brief screener and mathematics achievement measured at the end of first grade. The following hypotheses were tested: children who started first grade with low numerical competencies remained low mathematics achievement at the end of first grade; and children who started with high numerical competencies, finished the first grade with high mathematics achievement. The results showed that ENC contributed to a significant amount of explained variance in mathematics achievement at the end of the first grade. Children with low numerical competencies performed lower than children with moderate and high numerical competencies. Findings suggest that ENC are meaningful for predicting first-grade mathematics difficulties.

## Introduction

Difficulties in mathematics are pervasive and can have lifelong consequences (Jordan, 2010). Androulla Vassiliou, Commissioner responsible for Education, Culture, Multilingualism and Youth, endorsed this argument saying that: “Competence in mathematics has been identified at EU level as one of the key competences for personal fulfillment, active citizenship, social inclusion and employability in the knowledge society of the 21st century. Concerns about low student performance, as revealed by international surveys, led to the adoption in 2009 of an EU-wide benchmark in basic skills” [Education, Audiovisual and Culture Executive Agency (EACEA P9 Eurydice), 2011, p.3].

Identifying these basic skills has been a mathematical cognition research concern for the past two decades, and some key findings relevant to later achievement in mathematics were pointed out by Alcock et al. (2016). For instance, longitudinal studies are necessary to investigate which early abilities predict later mathematics achievement, and it is crucial to map predictors of mathematical competence to develop valid and reliable measures and to design effective interventions. Screening and targeted intervention would allow young children at risk for failure in mathematics to be identified and supported at early ages (Jordan et al., 2010).

The present study attempts to contribute to this research which aims to analyze the correlation and predictive evidence between early numerical competencies (ENC) (which are pointed out as one of the foundations of mathematical competence) and mathematics achievement at the end of first grade. We expect to enhance the transcultural evidence and consistency of international studies that analyzed and verified the predictive relation between these two variables in different contexts, in this case, in the Portuguese setting.

Our study may not only contribute to the international literature, as mentioned above, but may also give theoretical support to national literature, due to the fact that the National Council of Education (Conselho National de Educação, 2015) reported the need for an early identification and intervention at the first signs of school failure, particularly in kindergarten and in the first years of schooling, to fight the national grade retention considered 10% in 2nd grade, and to combat the difficulties that 4th grade students showed in mental calculation, in performing arithmetic operations, and in other mathematics areas, such as geometry.

In this paper, we refer to the ENC as a set of symbolic numerical abilities (also known as symbolic number sense) received from cultural and learning inputs, which may depend on the development and the integration of multiple basic cognitive abilities (Dehaene, 2011), such as working memory. Hornung et al. (2014) revealed that non-verbal number sense and working memory are central buildings blocks for developing ENC in kindergarten, and ENC is a key competence for first grade mathematics achievement.

Although there is no one definition of ENC, several researchers agree that this set of symbolic competencies in the 4- to 6-year-old range refers to: (a) counting in a small set of objects; (b) number identification; (c) making relations about numbers (e.g., 4 is closer to 3 than to 6) and their magnitudes (e.g., 5 is more than 3); and (d) basic arithmetic operations, which means transforming sets of numbers by adding or taking away items (e.g., 3 and 2 makes 5, and taking away 2 from 5 is 3) (Griffin and Case, 1997; Gersten and Chard, 1999; Berch, 2005; Jordan et al., 2008).

### Counting

To represent larger sets precisely, children need to learn how to count (Jordan and Levine, 2009). With the introduction of a verbal representation – the number word, counting is considered a symbolic numerical competence, which includes oral and object counting. Children can often count to 10 (reciting 1, 2, 3, 4, 5…), but they may not understand what numbers represent (Bermejo et al., 2004). To do so, children need to gradually bring meaning to counting (Dehaene, 2011). Meaning to counting signifies that numbers in the counting sequence have larger quantities than earlier numbers (e.g., n; n+1; (n+1) +1, etc.) and numbers have exact magnitudes (Sarnecka and Carey, 2008). For small sets (i.e., sets of 3 or less), children first map number words through subitization (Le Corre and Carey, 2007). For larger sets, counting is usually needed to determine the cardinal value. During preschool and kindergarten, most children learn to enumerate sets in a stable order (e.g.,1, 2, 3, 4, 5) using one-to-one correspondence between the number and the object, and come to realize cardinality, which means that the last number indicates the number of objects in a set (Gelman and Gallistel, 1978). Understanding these counting principles allows children to enumerate any type of sets (e.g., heterogeneous or homogeneous) in any direction (e.g., left to right or right to left and so forth). Children also use meaning to counting to construct a linear representation of numerical magnitudes (also known as mental number line), which allows them to compare numbers, learn how to place values in our base-10 number system, and manipulate sets through addition and subtraction, with and without object representations (Levine et al., 1992).

### Number Identification

Identifying numbers implies the ability to identify or recognize a number symbol (e.g., 13) or number symbols combined to represent any number (e.g., 128) presented as a visual stimulus. To answer the question “What number is this?” children must learn number-words using long-term memory.

### Comparing Numbers

Comparing numbers on the most basic level implies that children look at two numbers (e.g., 4 and 9) and answer the question, “Which is bigger?”(9) or “Which is smaller?”(4). Preschool students, when presented with two non-symbolic sets for comparison, often do not count when comparing the two sets. Typically, students rely on visual (i.e., non-symbolic) inspection (Zhou, 2002). This helps students only for a while, generally when numbers are between 1 and 3, after which they rely more on the system of approximate magnitudes to make judgments of small quantities larger than 3 (Xu and Spelke, 2000), using the mechanism to represent approximate quantities. At 6 years of age, students integrate counting schemes that allow them to develop a numeric mental line (Siegler and Booth, 2004). It is this mental line that will give them better knowledge of “quantitative words” (Griffin, 2002), and to understand that the numbers themselves in this numerical sequence have distinct magnitudes numbers (e.g., 5 is a successor number and greater than 4 and predecessor number, less than 6). Understanding that numerical sequence is becoming larger and more inclusive than the preceding numbers, the student begins to command the identification of successor numbers and predecessors (Le Corre and Carey, 2007, Sarnecka and Carey, 2008). Students also need to discriminate and relate quantities using symbolic mathematical processes, such as the use of numerals and cardinal numbers. For instance, knowing that eight (8) is bigger (>) than five (5), six (6) is smaller (<) than nine (9), or that 5 is closer to 6 than to 7 (Case and Griffin, 1990). Studies with adults showed that students have an easier time discriminating between quantities that are much farther apart (e.g., 9 and 2) than those that are closer in magnitude (e.g., 9 and 8) (Murray and Mayer, 1988).

### Basic Arithmetic Operations

Adequate counting, comparing, and symbol knowledge skills are necessary to carry out most addition and subtraction problems presented to students in early elementary school (Powell and Fuchs, 2012). To learn about the addition and subtraction (i.e., basic facts), students often work on simple problems with manipulatives. With practice, students rely less on manipulatives and more on their fingers for counting (Groen and Resnick, 1977). Because counting is often involved in solving basic addition and subtraction, counting skills are important (Baroody et al., 2009). Most of the time, young students use counting by ones as their default counting mechanism, and then counting by twos or utilizing subitizing skills (Camos, 2003). Students then move from counting to solving basic arithmetic operations using reasoning strategies (e.g., the friends of 10’s) or from memory (e.g., fact retrieval). Mastery of and fluency in numerical calculations (fact retrieval and computation fluency) is the end goal of basic arithmetic operations, and it is considered a necessary foundation at many levels – from solving simple word problems, to calculating with fractions, decimals and percentages, to solving algebraic equations or even to calculating with basic geometry (Jordan et al., 2007, 2008).

Some studies indicate that ENC, especially with respect to basic arithmetic operations, are closely tied to the mathematics curriculum in elementary school. For instance, the results of the Geary et al. (2000) study, showed that weak numerical competencies are associated with characteristics of mathematics learning difficulties, such as poor counting procedures, slow fact retrieval, and inaccurate computation. Therefore, inaccurate and dysfluent numerical calculation, considered a signature characteristic of students with learning difficulties in mathematics, is identified as a fundamental weakness in numerical competencies (Gersten et al., 2005; Jordan et al., 2008). Moreover, Locuniak and Jordan (2008) assessed ENC using symbolic and non-symbolic tasks in 198 kindergarten children, and again in second grade on a calculation fluency measure. Students scoring below the 25th percentile at the beginning of kindergarten were designated at risk for poor mathematics development. The results showed that approximately 52% of children with difficulties in fluency in numerical calculations were identified as at-risk students.

These findings suggested that ENC is a crucial predictor of early mathematics achievement. Children who started first grade with advanced ENC will consequently progress faster in arithmetic and generally in symbolic numerical tasks in the first grade (Bartelet et al., 2014). Hornung et al. (2014), using structural equation modeling and mediation analyses, suggested that ENC (verbal counting, dot counting, and Arabic number comparison, measured in kindergarten) is a critical mediator of the relationship between kindergarten non-symbolic domain-specific (i.e., approximate estimation of quantities) and domain-general (i.e., working memory) predictors and first grade mathematics achievement. They found that non-symbolic domain-specific abilities and working memory turned out to be critical for ENC in kindergarten, but not for first grade mathematics achievement after ENC was taken into account. In line with these studies, Jordan et al. (2007) indicate that the development and performance of counting, number identification, number comparison and basic arithmetic operations in kindergarten children explain 66% of mathematics achievement at the end of first grade.

### Purpose and Research Questions

The present study aims to understand to role of ENC in mathematics achievement in Portuguese first school children. The main purpose is to analyze the correlation and predictive evidence between ENC and mathematics achievement at the end of first grade. The following research questions were considered:

(1) What is the correlation between ENC and mathematics achievement at the end of first grade?;
(2) Do ENC at the point of school entry predict mathematics achievement at the end of first grade?;
(3) What is the relation between low, moderate, and high numerical competencies and mathematics achievement? In this research question, two hypotheses were tested: (a) children who started first grade with low numerical competencies remained low mathematics achievement at the end of first grade; (b) children who started with moderate numerical competencies, finished the first grade with moderate to higher gains on mathematics achievement; (c) children who started with high numerical competencies, finished the first grade with high mathematics achievement.

## Materials and Methods

The NSB – Number Sense Brief Screener (Jordan et al., 2008) was adapted to the Portuguese population (*n* = 2246), and psychometrics properties analyzed (Marcelino et al., 2012; Marcelino, 2015). Children were evaluated at the beginning of first grade (defined as a status point) and mathematics outcomes were obtained adding a formal mathematics achievement test (MSE – Math Summative Evaluation), the psychometric properties of which were also analyzed. Correlation and regression data analyses were used to evaluate the association between NSB and MSE.

Children were split into the low, middle, and high grounds based on the normative values of NSB. Children were assigned to the low numerical competence group (A) when they performed at or below the 25th percentile; average numerical competence (B) and high numerical competence (C) children’s groups were defined as performing on the 25–75th range, and above the 75th percentile, respectively.

### Participants

The participants were children who, after kindergarten, attended the first grade (first year of formal schooling in Portugal) in four elementary public schools. The average age was 6.37 (*SD* = 0.74); 71 of the 123 students were boys and 52 were girls. All children were taught mathematics with the same formal curriculum mathematics contents.

The public schools are located in a residential area of western Lisbon with different background characteristics concerning the level of education and socioeconomic level of the local population. The southern part has a family-unit urbanization with houses, where the level of education and the socioeconomic level is medium-high. The northern part has buildings (median five stories) with a medium to low socioeconomic level.

Among the demographic characteristics of the three working samples, Group C (high numerical competence) has more boys than girls, and the parents’ education is higher when compared to Group A (low numerical competence).

The demographic information of the overall sample and the three working sample is portrayed in **Table 1**.

**Table 1:** Demographic information for participants.

	NSB	Group A	Group B	Group C
**Gender**				
Male	57.70	53.70	53.80	71.40
Female	42.30	46.30	46.20	28.60
**Parent’s education**				
Primary education	6.80	10.30	5.00	5.30
Secondary education	52.50	61.90	54.60	42.90
Higher education	40.70	27.80	40.40	51.80

*Each value corresponds to the value’s percentage of the total. Parents’ education level refers to primary (up to fourth grade), secondary (up to twelfth grade), and higher education (concluded). NSB, number sense brief screener overall; Group A, low numerical competence; Group B, moderate numerical competence; Group C, high numerical competence (C). NSB (*n* = 123); Group A (*n* = 43); Group B (*n* = 52); Group C (*n* = 28).*

### Materials

#### Number Sense Brief Screener (NSB)

The NSB is a shortened version (Jordan et al., 2008) of the Number Sense Battery developed by Jordan et al. (2006). It is a number sense assessment tool for identifying children at risk for mathematics difficulties. The items assess symbolic numerical competencies – counting knowledge and principles, number identification, number comparison, non-verbal calculation, story problems, and number combinations. The NSB can being applied to 4- to 6-year-old children. The total possible score on the NSB is 33 points in a dichotomous scale (1 = correct; 0 = incorrect). The NSB is reliable, with a Cronbach alpha of 0.84 at the beginning of first grade, with a raw score mean of 21.83 (Jordan et al., 2008, 2010).

The NSB raw score means for the Portuguese population is 22.87 points (Marcelino, 2015) (scale in the range 0–33). The psychometric analysis indicated that NSB is reliable with a coefficient Cronbach alpha of 0.87 (*n* = 2246) at the beginning of first grade. The measure shows homogeneity between items, with an acceptable item-total mean correlation of 0.69.

The composite achievement score in NSB (NSB overall) was the combined raw scores for seven subtests assessing the following ENC:

##### Counting (3 items)

After the child finished counting a set of five stars, the examiner asked the following question: “How many stars were on the paper you just saw?” Counting sequence also included counting to 30.

##### Counting principles (4 items)

To assess counting principles, children were asked to recognize correct, incorrect counts (e.g., counting the first object twice), and correct unusual counts (e.g., counting from right to left or counting the yellow dots first and the blue dots afterward).

##### Number identification (4 items)

Children were asked to name a visually presented number (e.g., 13) with the question “What number is this?”

##### Number comparisons (7 items)

Children were asked to make numerical magnitude judgments in three different ways: (a) given a number (e.g., 7), children were asked what number comes after the given number and what number comes two numbers after the given number; (b) given two numbers, children were asked to indicate which of two numbers was bigger or smaller; (c) and given three number (e.g., 6, 2, and 5), each placed on the point of an equilateral triangle, children also were asked to identify which number was closer to the target number.

##### Non-verbal calculation (4 items)

On non-verbal calculation (presence of objects but without verbal stimuli) the examiner showed a set of chips, covered them, and then performed the addition or subtraction transformation (by removing or adding chips). Children were then asked to indicate how many chips were then under the cover.

##### Story problems (5 items)

On story problems (objects referents with verbal stimuli), children were orally phrased to three addition and two subtraction story problems and asked to solve them (e.g., Jose has 3 cookies. Sarah gives him 2 more cookies. How many cookies does Jose have now?).

##### Number combination (6 items)

On number combination (no object referents with verbal stimuli), children were asked to solve four addition and two subtraction computations (e.g., How much is 3 and 2?).

#### Mathematic Summative Evaluation (MSE)

Achievement in mathematics was assessed using a formal Portuguese school evaluation of mathematics. The MSE was applied by teachers to measure mathematics achievement at the end of first grade in the classroom for 1 h and a half. The measure is based on 25 items. Results were normalized to a total of 100%. The items included four subtests using the quantities 1 up to 99.

The composite achievement score (MSE overall) was the combined raw scores for subtests assessing counting and arithmetic operations, place value, applied problems and basic geometry.

The psychometric analysis showed that MSE is reliable with an internal-consistency reliability of 0.87 (*n* = 119). Subtests coefficients alpha were also analyzed, respectively, counting and operations (α = 0.729), place value (α = 0.764), applied problems (α = 0.804) and, forms and spatial (α = 0.669). The measure shows homogeneity between items, with an acceptable item-total mean correlation of 0.71.

##### Counting and operations

Children were asked to solve exercises concerning object counting to 10, counting by fives up to 30, counting money up to 10 (e.g., 1€ + 2€ + 5€), ordering numbers up to 99, adding and subtracting facts up to 99 (e.g., 15+25 = ___, 50-40 = ___) and missing values with addition facts (e.g., 80 = 40+___).

##### Place value

The tasks were related to units and tens identification with abacus and Cuisenaire rods models. Children also had to identify predecessor and successor numbers up to 99 (e.g., __ 49 __), and to identify several numbers on a number line (with interval from 10 to 90) partially filled with numbers.

##### Applied problems

Children were asked to solve three addition and subtraction word problems up to 50. Another word problem concerned the interpretation and counting data in a 2×2 table.

##### Forms and spatial

The tasks were related to geometry (e.g., to identify basic geometric figures), time (e.g., to identify the days of the week) and spatial sense (e.g., to design a symmetry figure).

### Procedure

This study was carried out in accordance with the recommendations of the National Direction for Education with written informed consent to contact the national groups of schools. After one national group of schools with four primary public schools located in Lisbon had given permission to apply the present study, the sample were collected with the parents’ written informed consent, with a notification that any elements of research will be covered by the anonymity of the participants.

Children were assessed individually with the NSB at the point of school entry by examiners who were fully trained in the testing procedures. The examiners were graduate students in Psychology and Education Sciences. Based on the NSB initial application protocol (Marcelino, 2015), the examiner read the questions to children individually while the children were seated next to him in a quiet room. Children’s mathematics achievement was assessed at the end of first grade by teachers in the classroom using a mathematics achievement formal test (MSE).

## Results

### Correlation Evidence between Early Numerical Competencies and Mathematics Achievement

To determine the association between ENC (assessed by NSB raw scores) and mathematics achievement (assessed by MSE raw scores), bivariate correlations were analyzed and are presented in **Table 2**. The correlation between NSB overall and MSE overall was moderate, positive and statistically significant (*r* = 0.57). Between NSB overall and MSE subtests, all the correlations were also moderate, positive and statistically significant, with the highest correlation to be found in spatial and forms and counting and operations (0.50 and 0.50, respectively).

**Table 2:** Correlations between early numerical competencies and dependent variables.

	MSE	CO	Pv	Ap	FS
NSB	0.57ˆ**	0.50ˆ**	0.47ˆ**	0.39ˆ**	0.50ˆ**
Counting	0.10	-0.03	0.07	0.08	0.19ˆ*
Counting principles	0.18	0.18	0.10	0.12	0.13
Number identification	0.42ˆ**	0.36ˆ**	0.34ˆ**	0.32ˆ**	0.28ˆ**
Number comparisons	0.37ˆ**	0.35ˆ**	0.32ˆ**	0.24ˆ**	0.34ˆ**
Non-verbal calculation	0.20ˆ*	0.18ˆ**	0.12	0.12	0.22ˆ*
Story problems	0.50ˆ**	0.46ˆ**	0.41ˆ**	0.34ˆ**	0.43ˆ**
Number combinations	0.49ˆ**	0.39ˆ**	0.42ˆ**	0.34ˆ**	0.47ˆ**

MSE, math summative evaluation overall; CO, counting and operations; Pv, place value; Ap, applied problems; FS, forms and spatial; NSB, number sense brief screener. *n =* 118. *^∗^p* < 0.05. ^∗∗^*p* < 0.01.

Regarding the NSB subtests and MSE overall, number identification (*r* = 0.42), story problems (*r* = 0.50) and number combinations (*r* = 0.49) were the highest correlation found, with the lowest correlations being counting and principles (*r* = 0.10 and *r* = 0.18, respectively).

### Predictive Evidence of Early Numerical Competencies in Mathematics Achievement

The main purpose of the study was to determine the contribution of the NSB in predicting mathematics achievement at the end of first grade. To accomplish this goal, students’ scores on the NSB were regressed on the outcomes of ASM overall. As we are conducting a simple regression analysis on two variables which, on a first approach, are linearly dependent, the major assumptions concerns normality and homoscedasticity. In this regard, normality P–P plots and Q–Q plots of the standardized residuals were conducted. Shapiro–Wilk test of normality of the standardized residuals reveals a significance of 0.043 which, *per se*, does not represents a big “violation.” On the other hand, the scatter-plot of the Standardized Residuals (Min. -2.20, Max. 1.85, *MD* = 0.000, *SD* = 1.000) vs. Standardized Predicted Value (Min. -2.21, Max. 1.71, *MD* = 0.000, *SD* = 0.996) is rather rectangular, indicating that behavior is not far from homoscedasticity. This is confirmed by the Breusch–Pagan test which gives χ^2^ (1) = 3.247 with a significance of 0.072.

**Table 3** presents the results for predicting on the MSE overall mathematics scores at the end of first grade. Results showed that the NSB was statistically significant to the prediction outcomes in MSE overall (*p* < 0.01). The NSB overall accounted for 33% of the variance in mathematics achievement scores.

**Table 3:** Variance explained and regression coefficients by early numerical competencies (assessed by NSB and subtests) in Mathematics Achievement (assessed by MSE).

	R^2^	B	Beta	*t*-value	*p*-value
NSB	0.33ˆ**	2.16ˆ**	0.57	7.53	0.00
Counting	0.01	5.59	0.10	1.06	0.29
Counting principles	0.03	5.73	0.18	1.96	0.05
Number identification	0.17ˆ**	6.99ˆ**	0.42	4.94	0.01
Number comparisons	0.14ˆ*	5.17ˆ*	0.37	4.32	0.02
Non-verbal calculation	0.04ˆ*	5.77ˆ*	0.20	2.14	0.04
Story problems	0.25ˆ**	6.47ˆ**	0.50	6.17	0.00
Number combinations	0.24ˆ**	4.87ˆ**	0.49	6.01	0.00

*MSE, math summative evaluation; NSB, number sense brief screener overall. ^∗^*p* < 0.05, ^∗∗^*p* < 0.01.*

Independent regressions of the different subareas of NSB vs. MSE were performed to recognize their relative importance on the prediction. Story problems (*R*^2^ = 0.25) and number combinations, (*R*^2^ = 0.24) accounted moderately for about 25% of the explained variance in MSE achievement. A stepwise regression analysis with all subareas of NSB showed that story problems by themselves accounted for 24.7% of the variance in mathematics achievement in first grade, and together with number identification accounted for 31%. Individual subareas of the NSB, such as counting and counting principles are not statistically significant in predicting mathematics achievement (*p* > 0.05).

### Differences between NSB Achievement Groups and Mathematics Performance

The differences between NSB outcomes groups (Group A, B, and C; low, moderate, high numerical competencies) allow us to test if children who started first grade with low numerical competencies remained low mathematics achievement at the end of first grade; and children who started with high numerical competencies finished the first grade with high mathematics achievement. Differences between NSB outcome groups were observed when mathematics achievements were measured at the end of first grade.

As data do not fit a normal distribution, a Kruskal–Wallis test was used to measure if differences between NSB groups’ means were statistically significant. In this regard, we found significant group differences (A, B, and C) in mathematics achievement scores at the end of first grade, χ^2^ (2) = 28.34, *p* < 0.001, with score means in MSE about 60% (Group A), 72% (Group B), and 84% (Group C). Group A had the lowest mathematics means score, and Group C the highest in MSE overall and subareas. Group A was the only NSB group achievement who performed below in MSE overall and subareas (**Table 4**).

**Table 4:** Mathematics achievement means score overall and subareas by NSB outcome groups.

	NSB	Group A	Group B	Group C
	*MD (DP)*	*MD (DP)*	*MD (DP)*	*MD (DP)*
NSB	22.42 (05.83)	15.35 (02.93)	23.80 (02.35)	29.60 (01.63)
MSE	70.39 (21.39)	57.49 (23.01)	71.66 (19.53)	83.63 (10.55)
Counting and operations	16.86 (05.84)	14.05 (05.71)	16.96 (05.22)	21.16 (03.31)
Place value	14.51 (04.54)	12.20 (05.23)	14.92 (03.98)	17.34 (02.04)
Applied problems	18.84 (10.00)	14.79 (10.87)	19.18 (09.36)	24.82 (06.01)
Spatial and forms	21.20 (06.30)	17.58 (06.63)	21.96 (05.92)	25.23 (02.61)

*MSE, math summative evaluation overall; *SD*, standard deviation, Group A (*n* = 43); Group B (*n* = 52); Group C (*n* = 28). NSB, number sense brief screener overall.*

## Discussion

Early numerical competencies are considered a foundational domain-specific cognitive factor in the development of mathematical competence, allowing students to make connections with mathematical relationships, principles and procedures. Doing so, students can learn with success advanced mathematics (Gersten et al., 2005). Measuring ENC as early as possible is important in predicting later mathematics achievement, and can identify students at risk for having future mathematics difficulties (Jordan et al., 2007; Powell and Fuchs, 2012).

The main purpose of this study was to better understand the predictive relationship between early numerical competence (or number sense) and mathematics achievement in Portuguese students. Specifically, we attempted to predict achievement in mathematics at the end of first grade by measuring early number competencies at the point of entering school (i.e., prior of formal education). We used two measures: NSB – Number Sense Brief Screener and MSE – Math Summative Evaluation in 123 children from an urban public-school setting.

The results indicated that numerical competencies (as assessed by NSB) had a moderate predictability for the performance of mathematics at the end of first year in Portuguese children. Jordan et al. (2007) found that the development and performance of counting, number identification, number relations and basic arithmetic operations in kindergarten children had a moderate to strong predictability of mathematics achievement at the end of first grade (Jordan et al., 2007, 2010, respectively). Our findings are in accordance with the results of Jordan and colleagues’ studies concerning ENC as an important predictor of the achievement in mathematics, which supports transcultural evidence.

Our findings also show a positive, significant and moderate correlation between numerical competencies (as assessed by NSB) at the beginning of first grade, and the performance of mathematics at the end of first grade. The results indicated that basic arithmetic operations had the highest correlation with mathematics achievement when compared to other individual subareas of NSB.

Consistent with these findings, Jordan et al. (2006) found strong and significant correlations between basic arithmetic operations (number combinations and story problems) and mathematics achievement. Children developed an understanding of basic arithmetic operations and how they related to one another in the preschool years. Throughout the first year, children with low numerical competencies had difficulties in performing basic arithmetic operations involving addition and subtraction facts. Our results support other studies suggesting that weaknesses in numerical competencies, particularly those related to basic arithmetic operations, underlie most mathematical learning difficulties (e.g., Landerl et al., 2004; Gersten et al., 2005; Geary et al., 2007).

As stated by Baroody et al. (2009) fluency in basic arithmetic operations appears to begin with and grows out of number competencies. As they develop numerical competencies in preschool and elementary school, children acquire abilities to perform basic computations quickly and proficiently. If they show low numerical competencies at the beginning of school, their abilities to perform basic computations are at risk for failure.

Our results do not indicate that counting skills by themselves underlie mathematical difficulties. Noticeably, there is almost no difference in object counting tasks in the three different NSB achievement groups when compared to other mathematics contents tasks. Jordan et al. (2007) found that, apart from counting and principals, the remaining individual subareas of NSB had good predictability in mathematics achievement (assessed by the WJMath; McGrew and Woodcock, 2001), at the end of first grade. Although counting is a mathematical formal task highly correlated to arithmetic operations, by itself it does not appear to be a good predictor in mathematics achievement.

Related to the third research question, the results indicated that children who started first grade with low numerical competencies remained low mathematics achievement at the end of first grade; and children who started with moderate and high numerical competencies, finished the first grade with moderate and high mathematics achievement, respectively. The significant group differences found allow us to assume that ENC are important for setting learning trajectories in mathematics (Mazzocco and Thompson, 2005; Jordan et al., 2007; Jordan and Levine, 2009).

## Summary and Concluding Discussion

With the methodology herein presented (short-term longitudinal study) we can predict, in a reasonable way, that numeracy indicators as well as number identification, story problems and number combinations measured at the point of school entry, predict later mathematical performance, specifically at the end of first grade. As a final note, with this study we cannot state that the explained variance of mathematics achievement is a consequence of a single variable – the ENC -, but based on other works its importance may be assumed. For instance, Jordan et al. (2010), using the same measure with general predictors such as language, visual-spatial relations and working memory, found that ENC additionally contributed to the variance explained in first grade.

As the absence of control variables is one of the limitations of the present study, future studies should compare NSB with other ENC screening tools to measure concurrent analysis, with the purpose of producing a coherent and predictive model between ENC and mathematics achievement.

From a conceptual point of view, these findings support the studies of Jordan et al. (2010) related to the NSB as a reliable early screening tool for identifying children at risk for failure in mathematics. It also gives good transcultural evidence of the potential importance of NSB in a country other than the United States. More importantly, the results of this study give a practical support to designing and applying effective early intervention programs in children with mathematical learning difficulties.

As mathematical thinking invades the daily activities of a young child, and poor mathematics achievement has been shown to be a major influence during school-age years, this work can contribute to identify Portuguese children who are at risk for failure in mathematics. Another implication for educational practice draws attention to the importance of an ENC screening tool for use in schools, clinics and other educational settings, with the purpose of helping children build numerical competencies as early as possible, giving them the background they need to achieve in mathematics during the first years of schooling.

## Ethics Statement

We declare that this study was carried out in accordance with the recommendations of the committee ethic of the MIME – Monotorização de Inquéritos em Meio Escolar, Direção-Geral de Educação, Ministério da Educação [MIME – Monitoring of School-based Inquiries, General-Direction of Education, Ministry of Education] with written informed consent to be used for all students of first-grade public schools and with anonymous reporting procedures. We also declare that the parents or legal guardian’s of all subjects gave written informed consent to carry out the current research in accordance with the Declaration of Helsinki. Finally, we state that the protocol was reviewed and approved by the Direção Geral da Educação.

## Author Contributions

Conceptualization: LM and ÓdS. Methodology: LM. Formal analyses: LM and AL. Writing-review and editing: LM, ÓdS, and AL.

## Conflict of Interest Statement

The authors declare that the research was conducted in the absence of any commercial or financial relationships that could be construed as a potential conflict of interest. The reviewer HSP and handling Editor declared their shared affiliation, and the handling Editor states that the process nevertheless met the standards of a fair and objective review.
